# Bromodomain Protein BRD4 Is Essential for Hair Cell Function and Survival

**DOI:** 10.3389/fcell.2020.576654

**Published:** 2020-09-08

**Authors:** Abhiraami Kannan-Sundhari, Clemer Abad, Marie E. Maloof, Nagi G. Ayad, Juan I. Young, Xue Zhong Liu, Katherina Walz

**Affiliations:** ^1^Department of Otolaryngology, Miller School of Medicine, University of Miami, Miami, FL, United States; ^2^The Dr. John T. Macdonald Foundation Department of Human Genetics, University of Miami, Miami, FL, United States; ^3^John P. Hussman Institute for Human Genomics, University of Miami, Miami, FL, United States; ^4^The Miami Project to Cure Paralysis, Department of Neurological Surgery, Miller School of Medicine, University of Miami, Miami, FL, United States

**Keywords:** Brd4, hearing loss, hair cell loss, hair cells, function, survival

## Abstract

Hair cells (HCs) play crucial roles in perceiving sound, acceleration, and fluid motion. The tonotopic architecture of the sensory epithelium recognizes mechanical stimuli and convert them into electrical signals. The expression and regulation of the genes in the inner ear is very important to keep the sensory organ functional. Our study is the first to investigate the role of the epigenetic reader *Brd4* in the mouse inner ear. We demonstrate that HC specific deletion of *Brd4 in vivo* in the mouse inner ear is sufficient to cause profound hearing loss (HL), degeneration of stereocilia, nerve fibers and HC loss postnatally in mouse; suggesting an important role in hearing function and maintenance.

## Introduction

The inner ear is a complex sensory organ that is involved in hearing and vestibular balance. The complex mechanical-electrical transduction process that takes place in the inner ear is not completely understood (Tekin et al., 2001). The cochlea comprises the organ of Corti, that contains specialized mechanosensory HCs that recognize mechanical stimuli and convert them into electrical signals. The expression and tight regulation of the genes in the inner ear is very important to keep the sensory organ functional, playing a major role in the development, differentiation and morphogenesis (Wu and Kelley, 2012). Epigenetic mechanisms have been implicated various developmental disorders (Ong and Corces, 2014). Although research on epigenetic pathways in the inner ear is very limited, a few studies have shown that it plays a crucial role in hair cell (HC) development, maintenance, function, survival and regeneration (Doetzlhofer and Avraham, 2017). Hearing loss (HL) is a common sensorineural disorder in humans. Approximately 70% of HL is congenital with an incidence of ∼1 in 500 in newborns in industrialized nations. Despite the number of genes that have been associated with HL, many cases remain unexplained. HL has been associated with genes that are involved in DNA methylation, histone modification and chromatin remodeling (Layman et al., 2015). BRD4 is a chromatin-binding protein that belongs to the family of bromodomain and extra terminal proteins (BET) that recognize and bind acetylated histones to facilitate gene transcription. It is amongst the most studied of the BET reader proteins. The domains of mammalian BET proteins are highly conserved, including mice (Wu and Chiang, 2007). Most of the current research on *BRD4* focuses its role in several cancers (Sanchez and Zhou, 2009; Wang and Filippakopoulos, 2015; Taniguchi, 2016). The importance of BET protein BRD4 has been investigated and characterized in the developmental processes of different cell types (Lee et al., 2017; Penas et al., 2019). *BRD4* has been associated with HL in previous studies (de Souza et al., 2018; Olley et al., 2018), despite which, its role in HC development has not yet been studied. In this study, we provide evidence that *Brd4* is necessary for the function and maintenance of the inner ear by studying a HC specific knock-out mouse model.

## Materials and Methods

### RNA Isolation

*Brd4*^+/+^ and *Atoh1-Brd4*^–/–^ cochlear sensory epithelium were isolated and immediately transferred to 250 μL TriZol (Invitrogen). These samples were subject to ultrasonic homogenization for complete dissociation and lysis. 200 μL chloroform was added to the lysate-TriZol mixture and mixed gently incubated at room temperature (RT) for 5 min and centrifuged at maximum speed for 20 min at 4°C. Clear supernatant (the aqueous layer) was transferred to fresh tubes and equal volume of 70% ethanol was added and mixed well. This mixture was transferred to spin columns from the RNeasy mini kit (Qiagen) and processed according to the manufacturer’s instructions (catalog #74104). RNA yield was quantified with a nanodrop spectrophotometer (ND-1000, Thermo Fisher).

### cDNA Synthesis and Real Time PCR (RT-PCR)

RNA was reverse transcribed into cDNA using qScript^TM^ XLT cDNA SuperMix, cDNA Reverse Transcription Kit (QuantaBio #95161-500). Adjusted volumes of RNA with a final concentration of 500 ng/μL was added into each reaction. RT-PCR was performed relative to *mGapdh* as control. Primers for the reaction was designed using Primer3^1^. *In silico* PCR was performed using the UCSC genome browser^2^ to confirm their specificity, amplicon size and detection of any primer dimers. RT-PCR Primers: Ex 5-6F: ATGGCAGAAGCTCTGGAGAA, Ex 5-6R: TTGGTACCGTGGATACACCA, mGapdh-F: ACCCA GAAGACTGTGGATGG, mGapdh-R: CACATTGGGGGTAGG AACAC.

### Cochlear Extraction and Sectioning

P0 mouse cochleae were dissected in 4°C 1× PBS and fixed overnight at 4°C in PBS containing 4% paraformaldehyde. 45 μm sections were obtained using a vibrotome (Leica VT 1200S).

### Generation and Genotyping of *Brd4* Conditional Knock Out Mice

**FIGURE 1:**
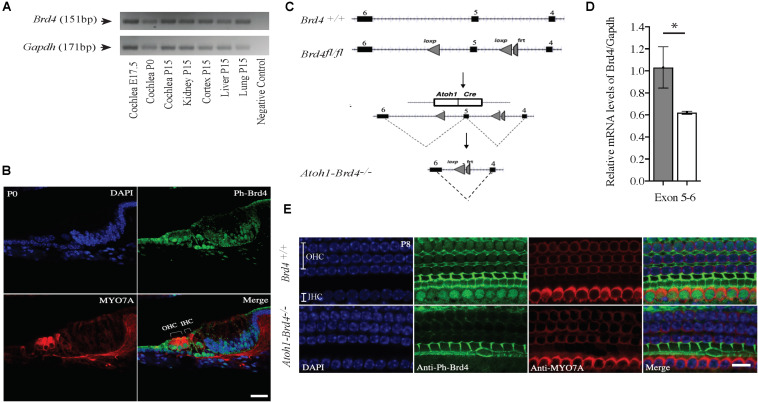
Mouse *Brd4* expression, generation, and confirmation of HC specific knockout. **(A)** Expression of *mBrd4* (Exon 5-6) in the cochlea during the different developmental stages (E17.5 to P15) is observed relative to *mGapdh*. **(B)** Immunostaining of *Brd4*^+/+^ section (*n* = 3) show *Brd4* expression (green) in nucleus of every cell-type in the inner ear as indicated by its colocalization with DAPI (blue, nuclei), including hair cells, labeled with HC specific Myo7a (red) (Scale = 10 μM). **(C)** *Brd4*^tm1c^ mice (referred to as *Brd4*^fl/fl^) were crossed with B6.Cg-Tg (Atoh1-cre) 1Bfri/J (The Jackson Laboratory Stock #011104) mice (referred to as Atoh1-cre), to obtain *Atoh1-Brd4*^–/–^ HC specific knockout and control littermates. **(D)** qRT-PCR results showed that *mBrd4* (Exon 5-6) levels were decreased significantly in (white) *Atoh1-Brd4*^–/–^ compared to (gray) *Brd4*^+/+^ samples (triplicates of each *n* = 3, *Brd4*^+/+^ and *n* = 4, *Atoh1-Brd4*^–/–^ samples). **(E)** Z-stack of P8 (*n* = 3) cochlea immunostained with Brd4 (green), the HC marker Myosin7a (red) and DAPI (blue, nuclei). Results showed Brd4 was absent in the IHCs and OHCs of the *Atoh1-Brd4*^–/–^ mice (Scale = 10 μM). (Standard *T*-test was performed to determine the significance of the experiment, ^∗^*p* < 0.05, ^∗∗^*p* < 0.01, ^∗∗∗^*p* < 0.001, ns-not significant).

Brd4^tm1a^ (EUCOMM)Wtsi (MGI ID: 4441798) heterozygous mice were obtained through the Knockout Mouse Project Repository (KOMP) at Baylor University from Dr. John Seavitt. These mice were bred to B6.Cg-Tg(ACTFLPe)9205Dym/J (The Jackson Laboratory Stock #005703) mice to remove the neomycin cassette and LacZ reporter gene to generate *Brd4*^tm1c^ heterozygous mice. The *Brd4*^tm1c^ mice (hereafter referred to as *Brd4*^fl/fl^) were crossed with B6.Cg-Tg(Atoh1-cre)1Bfri/J (The Jackson Laboratory Stock #011104) mice, hereafter referred to as Atoh1-cre, to obtain conditional cre expression in the HCs (Figure 1C). The mice were bred accordingly to generate *Brd4*^fl/fl;Atoh1–cre+/–^ (referred to as *Atoh1-Brd4*^–/–^) and control littermates. Tails were collected and labeled for gDNA extraction. They were incubated overnight at 56°C in 500 μL of TEL buffer and Proteinase K (20 mg/ml). The samples were centrifuged at maximum speed for 10 min. The supernatant was transferred to fresh tubes and DNA was precipitated using 100% Ethanol. The samples were centrifuged again at maximum speed for 5 min to pellet the DNA, then washed again with 70% ethanol for 2 min. The pellet was air dried for 20–30 min and dissolved in 150 μL of TE buffer (10 mM Tris–HCl pH 8.0, 0.1 mM EDTA pH 8.0). Standard PCR reactions were used to genotype the mice. Genotyping primers: Brd4-F-TCAGAGTGCTAGGGATTACAGG, Brd4- R- CTAGTTACAACGGCTTCTGTC, Cre-F-AGAACCTGAAGAT GTTCGCG, Cre-R-GGCTATACGTAACAGGGTGT.

PCR conditions: Initial Denaturation: 94°C for 4 min; Annealing: 94°C for 30 s, 60°C for 30 s, 72°C for 1 min repeated for 35 cycles; final extension: 72°C for 7 min; hold at 4°C ∞.

### Quantitative Real Time PCR (qRT-PCR)

Quantitative real time PCR (qRT-PCR) was performed using 2x Taqman Gene Expression Master Mix (Applied Biosystems/Invitrogen #4369016) with Taqman probes designed with the Taqman gene expression assay tool. Data were collected using the QuantStudio^TM^ 6 Flex Real-Time PCR System (Applied Biosystems/Invitrogen 4485694). Data analysis was done to asses fold change in gene expression with the comparative cycle threshold (ΔΔCt) method relative to housekeeping gene *Gapdh* cycle threshold values. Results were compared to gene expression in *Brd4*^+/+^ samples. qRT-PCR primers: Assay ID: Mm00480392_m1; Brd4 Ex5-6, FAM- MGB, Assay ID: Mm99999915_g1; Gapdh Ex 2-3; VIC-MGB.

### Section and Whole Mount Immunostaining

Sections from P0 and whole cochlear tissues collected from P8, P14, P16 and P21 *Brd4*^+/+^ and *Atoh1-Brd4*^+/–^ mice were fixed and immunolabeled. The tissues were permeabilized with 0.5% for 20 min, blocked for 1.5 h with a combination of 5% BSA and 0.1% Triton X-100 and incubated overnight at 4°C with primary antibodies. Immunolabeling of the primaries were done using secondary antibodies for 1.5 h at RT. The samples were then washed and incubated with DAPI (Calbiochem) for 5 min at RT. Specimens were washed with PBS and mounted in fluorescence mounting medium (Dako, # S3023). Preparations were imaged using a 63× objective with a Leica SP5 Inverted Confocal. Primary antibodies used are as follows: (1) Brd4 visualization: Rabbit anti-phospho-Brd4-S492/494 (1/50, Millipore, #ABE1453), (2) HC specific marker: Mouse monoclonal anti-Myo7a (1/400, Developmental Studies Hybridoma Bank at the University of Iowa, #138-1), (3) Neuronal fibers visualization: chicken polyclonal anti-Neurofilament (NF) (1/300, Millipore, #AB5539), (4) Synaptic ribbon visualization: mouse monoclonal Anti-Ctbp2 (1/300, BD Biosciences, #612044) and rabbit polyclonal anti-Glur2 (1/300, Millipore, # 07-598). Secondary antibodies used are as follows: (1) anti-rabbit Alexa Fluor^®^ 488 (1/400, Invitrogen, # A32731), (2) anti-chicken Alexa Fluor^®^ 488 (1/300, Invitrogen, # A32931), (3) anti-mouse Alexa Fluor^®^ 488 (1/400, Invitrogen, # A32723), (4) anti-rabbit Alexa Fluor^®^ 568 (1/400, Invitrogen, # A-11008), (5) anti-mouse Alexa Fluor^®^ 568 (1/400, Invitrogen, # A-21124), (6) Alexa Fluor^®^ Phalloidin 568 (1/100; Invitrogen, #A12380).

### Scanning Electron Microscopy (SEM)

P8 mouse cochleae from *Brd4*^+/+^ and *Atoh1-Brd4*^–/–^ were fixed in 3% glutaraldehyde in PBS overnight at 4°C. Samples were rinsed with PBS twice to remove the fixative well. Samples were fixed in 1% Osmium Tetroxide (OsO4) between 30 and 60 min, rinsed with distilled, de-ionized water for 10 min (2×), followed by treatment in 0.5% thiocarbohydrazide for 15–20 min. The process was repeated until the last OsO4 treatment was complete. The samples were rinsed well with water to remove any chemical residues and dehydrated in absolute EtOH serially from 50%, 70% up to 100% for 10 min each. The samples were then treated with HMDS for 5 min. Air contact was avoided to ensure there was no sample re-hydration. Once they were dried, the samples were mounted on scanning electron microscopy (SEM) stubs, the cochlea facing up and kept in a vacuum chamber till the time they were sputter coated with Palladium (Pd) (60:40) using Edwards S150B unit at 750 V and 40 mA for 30 min. Samples were imaged using an XL-30 field emission scanning electron microscope.

### Audio Brainstem Response (ABR) and Distorted Product Otoacoustic Emission (DPOAE) Evaluation

Mouse (*Brd4*^+/+^ and *Atoh1-Brd4*^+/–^) audio brainstem responses (ABRs) and distorted product otoacoustic emissions (DPOAEs) were conducted using a Smart EP Universal Smart Box (Intelligent Hearing Systems, Miami, FL, United States). ABR stimuli were 0.1 ms duration clicks or 0.1 ms duration pure tone pips presented at frequencies 8, 16, and 24 kHz. Click stimuli were enveloped with a rectangular window, while pips were enveloped with a Blackman window. Stimuli began at 20 dB SPL amplitude and increased at 10 dB steps up to 100 dB SPL. A total of 600 sweeps were averaged for each frequency and amplitude. ABR thresholds were determined for each stimulus frequency by identifying the lowest intensity producing a recognizable ABR pattern (at least two consistent peaks above the baseline). For DPOAEs, specific acoustic stimuli were delivered monaurally through plastic tubes channeled from high-frequency transducers. Five hundred and twelve sweeps of DPOAE at frequencies 2f1–f2 were recorded in response to two level primary tones (55 and 65 dB, respectively), f1 and f2, with f2/f1 = 1.20 and frequencies, √(*ff1* × *ff2*) ranging from 8 kHz to 24 kHz. *T*-test was performed for significance of experiment.

## Results

### *Brd4* Is Expressed in the Mouse Cochlea

In order to understand the spaciotemporal expression of the *Brd4* in the inner ear (IE), we analyzed its expression in wild type (WT) mice by RT-PCR and immunofluorescence. Expression of *Brd4* was studied by RT-PCR (Figure 1A) using mRNA obtained from mice cochlear tissues collected at embryonic stage E17.5, and postnatal stages P0 and P15. *Brd4* was detected in the *Brd4*^+/+^ mouse cochlea at E17.5, P0 and P15. Immunostaining (Figure 1B) of cochlear sections of P0 mice showed ubiquitous localization of phosphorylated-Brd4 (green) in the nuclei of cells, including HCs, identified by the specific marker Myo7a (red).

### Generation of HC-Specific *Brd4* Knock-Out Mice

To bypass embryonic lethality (Houzelstein et al., 2002) and investigate the function of *Brd4* in HCs, *Brd4* conditional knock-out mice were successfully generated as previously described (Penas et al., 2019; Figure 1C). *Brd4*^fl/fl^ were crossed with Atoh1-cre mice, that expressed Cre in developing HCs at approximately E13.5 (Woods et al., 2004) to obtain cell specific expression of cre in the HCs. The mice were bred accordingly to generate HC specific knockout *Atoh1-Brd4*^–/–^ and control littermates. In order to confirm the decrease in *Brd4* expression in *Atoh1-Brd4*^–/–^ mice we performed qRT-PCR using inner ear cDNA from P3 mice as template. A significant decrease in the levels of *Brd4* mRNA in *Atoh1-Brd4*^–/–^ mice compared to *Brd4*^+/+^ was observed (Figure 1D) and between *Brd4*^fl/fl^ and *Atoh1-Brd4*^–/–^ while there was no significant decrease observed between *Brd4*^+/+^ and *Brd4*^*fl/fl*^ (Supplementary Figures 1A,B). To visualize the HC specific deletion of *Brd4* in *Atoh1-Brd4*^–/–^ mice, we performed whole mount immunostaining on P8 cochlea. As expected, a clear deletion of *Brd4* in the outer hair cells (OHCs) and inner hair cells (IHCs) of the *Atoh1-Brd4*^–/–^ mice compared to *Brd4*^+/+^ was observed (Figure 1E).

### Auditory Neuropathy in HC-Specific *Brd4* Knock-Out Mice

**FIGURE 2:**
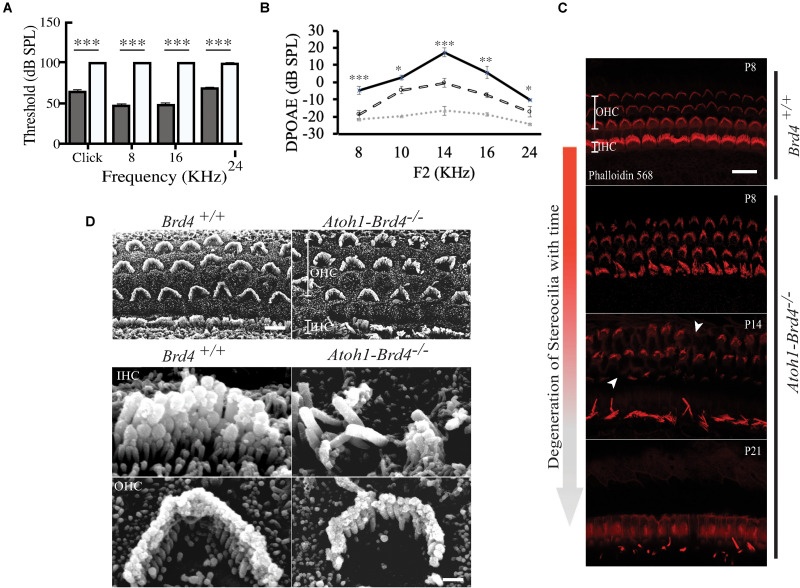
Auditory Neuropathy and Stereocilia degeneration. **(A)** The ABR thresholds for click response in (gray) *Brd4*^+^*^/^*^+^ mice (*n* = 4) were ∼65 dB SPL and ∼100 dB SPL in (white) *Atoh1-Brd4*^–/–^ (*n* = 5). Similarly, for the pure-tone frequencies, *Atoh1-Brd4*^–/–^ mice had significantly elevated ABR thresholds (∼100 dB SPL) compared with control mice. **(B)** The DPOAE recordings of (dashed white line) *Atoh1-Brd4*^–/–^ (*n* = 5) mice showed reduced amplitudes, from low to high frequencies (8–24 KHz) compared to (black line) *Brd4*^+/+^ group (*n* = 5). Noise floor is indicated with a dotted gray line. (Standard *T*-test was performed to determine the significance of the experiment, ^∗^*p* < 0.05, ^∗∗^*p* < 0.01, ^∗∗∗^*p* < 0.001, ns-not significant). **(C)** Z-stack of mouse cochleae (P8, *n* = 3; P14, *n* = 1; P21, *n* = 3) counterstained for actin filaments (red) (scale: 20 μm) showed degeneration of stereocilia over time between P8 and P21 in *Atoh1-Brd4*^–/–^. The white arrow heads represent the missing bundles of stereocilia. **(D)** SEM images of P8 (*n* = 2) mouse cochleae processed using the O-T-O-T-O showed show the difference in the structural integrity of the stereocilia between *Brd4*^+/+^ and *Atoh1-Brd4*^–/–^ (Scale: 10 μm upper panel; 2 μM lower panel).

To assess whether the cell-specific deletion of *Brd4* affected the hearing capacity we measured ABRs and DPOAEs. At P21, the ABR thresholds for click response in *Brd4*^+/+^ mice was ∼65 dB SPL (decibel sound pressure level) whereas in *Atoh1-Brd4*^–/–^ it was ∼100 dB SPL. Similarly, for the pure-tone frequencies, *Atoh1-Brd4*^–/–^ mice had significantly elevated ABR thresholds (∼100 dB SPL) compared with control mice (Figure 2A). Thus, the *Atoh1-Brd4*^–/–^ mice were profoundly deaf, indicating the important role of *Brd4* in HCs. The DPOAE recordings (Figure 2B) produced by OHCs in the *Atoh1-Brd4*^–/–^ mice had reduced amplitudes compared to the control group at P21; clearly suggesting a functional defect of OHC in the mutant mice. To exclude the effect of the floxed allele the hearing phenotype for *Brd4*^fl/fl^ mice was tested., A significant difference in the ABR and DPOAE was observed between *Brd4*^fl/fl^ and *Atoh1-Brd4*^–/–^ while there was no significant decrease observed between *Brd4*^+/+^ and *Brd4*^fl/fl^ (Supplementary Figures 1C–F); indicating that the floxed allele alone is not responsible for the HL observed in the HC-specific *Brd4* knock-out mice.

### Stereociliary Structure, Innervation Pattern, and Ribbon Synapses of Hair Cells Are Disrupted in HC-Specific *Brd4* Knock-Out Mice

**FIGURE 3:**
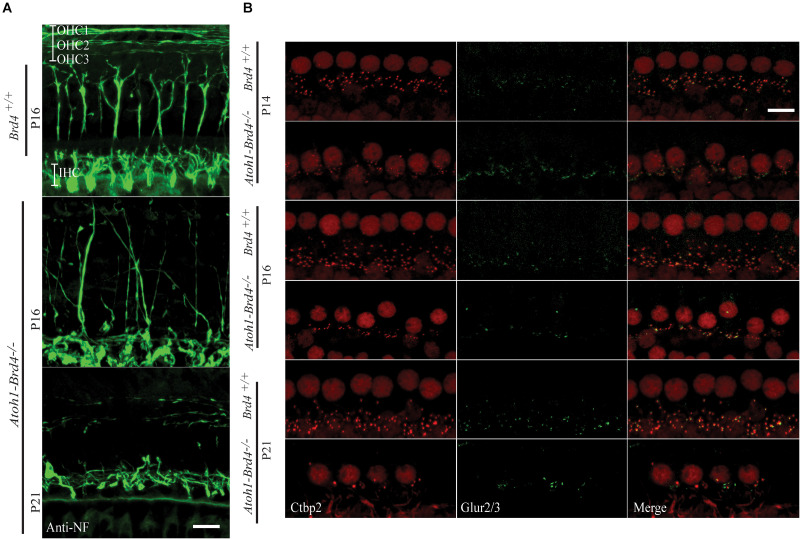
Innervation defects and degeneration of ribbon synapses. **(A)** Z-stack projections showing neurofilament fiber bundles with improper fasciculation and eventual degeneration in whole mount mouse cochleae *Atoh1-Brd4*^–/–^. Nerve fibers were immunolabeled with an antibody against neurofilament heavy chain (green) (bar scale: 20 μm). **(B)** Z-stack projections of mice IHCs stained for anti-RIBEYE/CtBP2 (red) and GluR2/3 (green). Reduced numbers of IHC paired CtBP2-GluR2/3 ribbons were observed starting at P14 and severe loss in synaptic ribbon formation was observed by P21 (bar scale: 10 μm).

To determine whether *Brd4* deletion cause morphological defects, we examined the HC in *Atoh1-Brd4*^–/–^ mice. Confocal microscopy and SEM images revealed that the stereocilia bundles in *Atoh1-Brd4*^–/–^ mice were abnormal compared to those in control mice. Confocal images of the middle region of cochlea showed morphological changes (Figure 2C) over time in between *Brd4*^+/+^ and *Atoh1-Brd4*^–/–^ starting with a mild disorganization in the stereociliary bundles at P8 in both IHCs and OHCs, to complete degeneration by P21. The SEM imaging confirmed the structural disorganization at P8 (Figure 2D). Morphological changes were also observed in the basal and apical regions (Supplementary Figure 2). In order to further examine the HL phenotype in the *Atoh1-Brd4*^–/–^ mice, we examined how the *Brd4* inactivation affects innervation of the inner ear. Confocal images of cochleae collected and immunolabeled with anti-neurofilament (NF) antibody, from *Brd4*^+/+^ and *Atoh1-Brd4*^–/–^ mice at P16 and P21 revealed severe disorganization of the neurofilament bundles and degeneration by P21 (Figure 3A). To determine if the synapses between HCs and the nerve terminals were affected, we collected the sensory epithelium from P14, P16, and P21 of *Brd4*^+/+^ and *Atoh1-Brd4*^–/–^ mice. When stained for Carboxy-terminal Binding Protein 2 (CtBP2), which labeled presynaptic ribbons found in IHC, and for GluR2/3, an AMPA receptor subunit that labeled postsynaptic glutamate receptors on afferent nerve terminals, reduced numbers of intact synapses in IHC were observed (Figure 3B).

### Severe HC Loss Is Observed in Brd4 Conditional Knock-Out Mice

**FIGURE 4:**
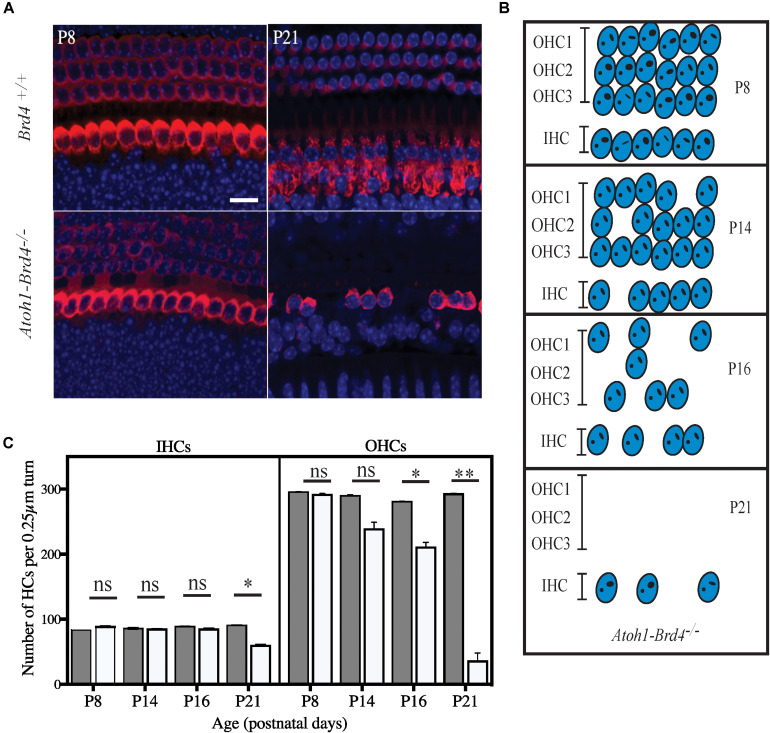
Severe hair cell loss. **(A)** Z-stack of *Atoh1-Brd4*^–/–^ mouse cochleae immunolabeled with HC marker Myo7a (red) and DAPI (blue) (scale: 10 μm) showed severe HC loss at P21 with more OHC death compared to IHCs. **(B)** A schematic representation of HC loss at different ages. **(C)** Graphical representation of the quantification of the number of OHCs and IHCs (per 0.25 μm turn of cochlea) of the cochlea at different developmental stages (white, *Atoh1-Brd4*^–/–^; gray, *Brd4*^+/+^). The error bars indicate the SEM (Standard *T*-test was performed to determine the significance of the experiment, ^∗^*p* < 0.05, ^∗∗^*p* < 0.01, ^∗∗∗^*p* < 0.001, ns-not significant).

We then examined whether HC loss occurred in the auditory epithelium of the *Atoh1-Brd4*^–/–^ mice. Confocal images of *Atoh1-Brd4*^–/–^ whole-mount specimen immunostained using HC marker Myo7a and DAPI to the stain the nuclei showed severe HC loss at P21 (Figure 4A). A schematic representation of HC death is shown in Figure 4B. Quantification of the total number of OHCs and IHCs was done through different postnatal developmental stages. No significant HC loss at P8 was detected. By P14, there was occasional OHC loss along the length of the cochlea, although the IHCs appeared largely intact. Beginning at P16, both the OHCs and IHCs underwent rapid cell death, OHCs more than IHC. By P21, there were very few OHCs in the cochlear epithelium. Although a majority of the OHCs were lost by P21 (∼90%; *p*-value < 0.01), a considerable number of IHCs remained (75%; *p*-value < 0.01). The quantification of the HC loss is shown in Figure 4C.

The loss of OHCs and IHCs across the different regions of the cochlea at P21 (base, middle, and apex) has been shown in Supplementary Figure 3.

## Discussion

An emerging paradigm in hearing biology is the role of transcriptional regulators in the structure and function of the inner ear. Increasing evidence suggests that epigenetic mechanisms are essential for establishing distinct chromatin states and cell-type specific gene expression patterns that play an important role in the development, function and maintenance of the inner ear. The inner ear sensory organ and the sensory neurons that innervate it, develop from the otic placode through a complex series of differentiation events that depend on the interplay of inductive signals and transcriptional activity. For example, the homeodomain transcription factors Otx2 and Gbx2 are critical for otic placode specification (Steventon et al., 2012). Epigenetic regulation of *GBX2* by *DNMT3A*, a *de novo* DNA methyltransferase (Roellig and Bronner, 2016) supports the involvement of epigenetic processes in normal development of the inner ear. Further, the histone demethylase KDM4B plays a critical role in regulating the expression of *Dlx3*, a member of the vertebrate Distal-less related (*Dlx*) homeodomain genes, required for otic invagination (Uribe et al., 2015). Haploinsufficiency in a member of the chromodomain helicase DNA-binding (CHD) family of ATP-dependent chromatin remodeling enzymes (*Chd7*), causes CHARGE Syndrome, which presents with inner ear defects (Vissers et al., 2004). Analysis of *Chd7* knockout mice revealed that CHD7 regulates the expression of vestibular regulatory genes in a gene dosage dependent manner (Hurd et al., 2010). In neuronal stem cells, it directly interacts with HMG-box transcription factor SOX2 and cooperatively regulates the expression of key developmental regulators including the Notch ligand Jagged1 (JAG1) and the transcription factor GLI3 (Engelen et al., 2011). JAG1 is an essential regulator of inner ear neuro-sensory development and is required for semicircular canal formation (Kiernan et al., 2001). Gli3 is necessary for the correct development of the apical region of the cochlea and its mutation causes Pallister-Hall syndrome which shows a prevalence of low-frequency HL (Driver et al., 2008). Interestingly, most CHD7 binding sites show features of gene enhancer elements. Atonal homolog 1 (Atoh1) is a transcription factor crucial for the generation of HCs and neurons in the inner ear. Dynamic changes in histone modifications at the *Atoh1* locus correlating with *Atoh1* expression reveal an epigenetic mechanism of *Atoh1* regulation underlying HC differentiation and subsequent maturation (Stojanova et al., 2015). Despite all this knowledge, the role of epigenetic regulation in the differentiation, maturation, and maintenance of auditory HCs still remains unclear.

Here we studied the role of BRD4, a member of the BET protein family that interacts with acetylated lysines on chromatin. BRD4 binds to hyperacetylated genomic regions that encompass promoters and enhancers and regulates transcription elongation by paused RNA polymerase II (Pol II). Regions of H3K27ac bound by BRD4 and mediator complex subunit 1 (MED1) have been defined as super-enhancers important to determine cell identity (Hnisz et al., 2013). Several lines of evidence suggested a role for *BRD4* in HL. It has been reported that haploinsufficiency of *BRD4* is associated with Cornelia de Lange syndrome (CdLS), a severe multisystem neurodevelopmental disorder presenting with HL as one of the clinical phenotypes (Olley et al., 2018). Similarly, a novel 2.52 Mb microdeletion at 19p13.12 that includes *BRD4* was also reported in a patient presenting with HL, using high resolution chromosomal microarray analysis (de Souza et al., 2018). In addition, BRD4 and P-TEFb were identified as WHSC1 interacting partners, and together they facilitate transcriptional elongation (Sarai et al., 2013) WHSC1-deficient mice fail to develop normal stereocilia hair bundles required for sound perception. The BET family is a distinct group of bromodomain proteins that in mammals includes BRD2, BRD3, BRD4 (ubiquitously expressed), and BRDT (testis specific). Brd2 and Brd4 have been extensively studied in the context of cell-cycle control and transcription elongation and activation and are known to be overexpressed in multiple tumor types. BET proteins promote the expression of oncogenes such as MYC, promoting tumorigenesis. The anti-proliferative effect of inhibiting BET prompted clinical trials for small molecule inhibitors of BET bromodomain proteins in cancer. JQ1 (thienodiazepine) is the one of the first published small molecules that binds competitively to bromodomains (Filippakopoulos et al., 2010). JQ1, along with other BET inhibitors, has been shown to have inhibitory effects in various human cancers and xenograft models (Doroshow et al., 2017). Although the therapeutic success of BET inhibition in clinical trials and animal studies generates enthusiasm, the potential toxicities at the effective doses remain indefinite at this time. Studies in mice have shown that *Brd4* binds to acetylated histone residues which are often modified in embryonic stem cells (ESCs) (Markowetz et al., 2010). In pluripotent cells, the *Brd4* and acetyltransferase complex regulate chromatin structure and mesoderm formation (Wu et al., 2018) and maintain pluripotency of ESCs (Gonzales-Cope et al., 2016; Fernandez-Alonso et al., 2017). *Brd4* has also been shown to have a role in learning and memory *in vivo* in adult animals (Korb et al., 2015; Sartor et al., 2015; Benito et al., 2017) by controlling the expression of these genes as well as synaptic receptor proteins related to memory formation (Korb et al., 2015). *Brd4* also plays a role in adipogenesis and myogenesis by specifically recruiting transcription elongation factors necessary to transcribe genes for brown adipocyte differentiation as well as myogenesis (Lee et al., 2017; Brown et al., 2018), and inducing differentiation in immune and epithelial cells in mice (Zhang et al., 2012; Devaiah et al., 2016).

In our study, we uncovered that deletion of *Brd4* in HCs of mice resulted in profound deafness, ultimately related with cellular death, supporting an important role for *Brd4* in the maintenance/function of HCs. Since *BRD4* has emerged as an attractive anticancer therapeutic target and its inhibition entered the clinical practice, this finding of BRD4 as a key molecule for the function of the inner ear prompts the study of potential effects of BET inhibition in audition and balance. The *Brd4* conditional knockout developed physiological as well a variety of morphological changes that occur in the cochlea. DPOAEs are used to evoke responses from the OHCs and based on the recordings or lack thereof, it is determined if OHCs are functional. As expected, the OHCs of the mutant mice in our studies were not functional as early as P21. ABRs are another type of hearing evaluation (tested across various frequencies) that specifically tests for the normal innervation of the ear. In this study, the mutants had a threshold of >100 dB, indicating that they did not hear at any frequency. This shows that the innervation in the mutant mice has possibly been damaged. The stereocilia bundles form the apical part of the HCs which are terminally differentiated and specialized (Raphael and Altschuler, 2003) and are extremely sensitive to mechanical displacement (sound). The stereocilia respond to sound by opening ion channels and depolarizing HCs, subsequently transferring the signal to the afferent neurons. This study revealed a progressive pattern of degeneration of these bundles, starting with a milder phenotype expressed at P8 and eventually complete loss of the cilia by P21. Integrity of spiral ganglion neurons and HC interactions are crucial for the mechanostransduction process in the inner ear. Upon investigation of the patterns of innervation and synapse formation, there was rapid degeneration of neuronal fibers and a gross reduction in the numbers of intact synapses. The innervation pattern appeared normal in *Brd4*^+/+^ as the fibers project toward their IHC and OHC targets and they cross the tunnel of Corti (TC) and fasciculate to form three discrete bundles. However, in the *Atoh1-Brd4*^–/–^, it can be observed that the fibers not only fail to fasciculate their target IHC and OHCs, they form loose or improper bundles and eventually degenerate. In the case of synapses, the number of CtBP2 puncta, GluR2/3-labeled terminals, and paired CtBP2-GluR2/3 ribbons were reduced starting at P14. By P16, a significant decrease in the synapse formation was observed and by P21 synapse formation was completely lost in IHCs. This indicates that Brd4 might play a role in regulating the synaptic ribbon formation and communication between SGNs and HCs, necessary for hearing. Finally, severe HC loss occurred in the auditory epithelium. In conclusion, we demonstrate with our study, a strong role for *Brd4* in the inner ear.

## Data Availability Statement

All datasets presented in this study are included in the article/Supplementary Material.

## Ethics Statement

The animal study was reviewed and approved by University of Miami Institutional Animal Care and the National Research Council (US) Guide for the Care and Use of Laboratory Animals.

## Author Contributions

AK-S was the primary author of this manuscript and has participated in the design of the study, acquisition, data generation and analysis, interpretation, and validation; also responsible for the preparation of this original manuscript. CA participated in the design and execution of the study and in revising the manuscript critically for important intellectual content. MM and NA participated in revising the manuscript. JY participated in the study design and revision of the manuscript. XL and KW were co-corresponding authors and participated in the supporting and supervising the study as well as critical revision of the manuscript. All authors contributed to the article and approved the submitted version.

## Conflict of Interest

The authors declare that the research was conducted in the absence of any commercial or financial relationships that could be construed as a potential conflict of interest.

## Footnotes

1 http://bioinfo.ut.ee/primer3-0.4.0/

2 https://genome.ucsc.edu/cgi-bin/hgPcr
