# Supplementary material for: Bromodomain Protein BRD4 Is Essential for Hair Cell Function and Survival
Source: Front Cell Dev Biol. 2020 Sep 8;8:576654. doi: 10.3389/fcell.2020.576654 (PMC7509448; doi:10.3389/fcell.2020.576654)
Supplement: Supplementary file 1 [file Image_1.pdf]

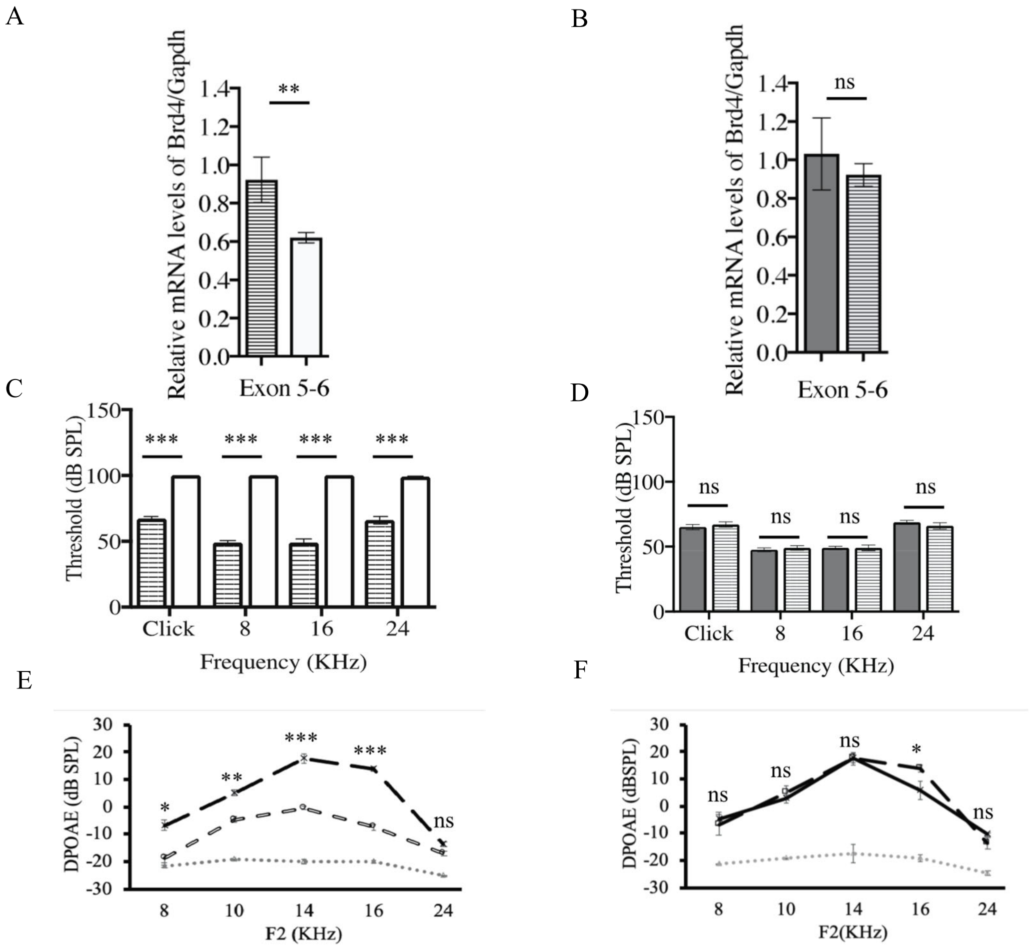

Supplementary Figure 1. *mBrd4* expression and auditory neuropathy (A) qRT-PCR results showed that *mBrd4* (Exon 5-6) levels were decreased significantly in (white) *Atoh1-Brd4*$^{-/-}$ compared to (striped) *Brd4*$^{fl/fl}$ samples (triplicates of each n=4, *Brd4*$^{fl/fl}$ and n=4, *Atoh1-Brd4*$^{-/-}$ samples). **(B)** qRT-PCR results showed that *mBrd4* (Exon 5-6) levels had no significant decrease in (grey) *Brd4*$^{+/+}$ compared to (striped) *Brd4*$^{fl/fl}$ samples (triplicates of each n=3, *Brd4*$^{+/+}$ and n=4, *Brd4*$^{fl/fl}$ samples) (Standard T-test was performed to determine the significance of the experiment, * $p<0.05$, ** $p< 0.01$, *** $p<0.001$, ns-not significant). **(C)** The ABR thresholds for click response in (striped) *Brd4*$^{fl/fl}$ mice (n=5) were ~ 67dB SPL and ~100 dB SPL in (white) *Atoh1-Brd4*$^{-/-}$ (n=5). For the pure-tone frequencies, *Atoh1-Brd4*$^{-/-}$ mice had significantly elevated ABR thresholds (~100dB SPL) compared with control mice. **(D)**The ABR thresholds for click response in (grey) *Brd4*$^{+/+}$ mice (n=4) were ~ 65dB SPL and ~67 dB SPL in (striped) *Brd4*$^{fl/fl}$ (n=5) with no significant difference. Similarly, for the pure-tone frequencies, they had similar thresholds with no significant difference. **(E)** The DPOAE recordings of (dashed white line) *Atoh1-Brd4*$^{-/-}$ (n=5) mice showed reduced amplitudes, from low to high frequencies (8KHz-24 KHz) compared to (dashed black line) *Brd4*$^{fl/fl}$ group (n=5). **(F)** The DPOAE recordings of (black line) *Brd4*$^{+/+}$ (n=4) mice showed no significant reduction in amplitudes, compared to (dashed black line) *Brd4*$^{fl/fl}$ group (n=5). Noise floor is indicated with a dotted grey line. (Standard T-test was performed to determine the significance of the experiment, * $p<0.05$, ** $p< 0.01$, *** $p<0.001$, ns-not significant).
