# Supplementary material for: Bromodomain Protein BRD4 Is Essential for Hair Cell Function and Survival
Source: Front Cell Dev Biol. 2020 Sep 8;8:576654. doi: 10.3389/fcell.2020.576654 (PMC7509448; doi:10.3389/fcell.2020.576654)
Supplement: Supplementary file 2 [file Image_2.pdf]

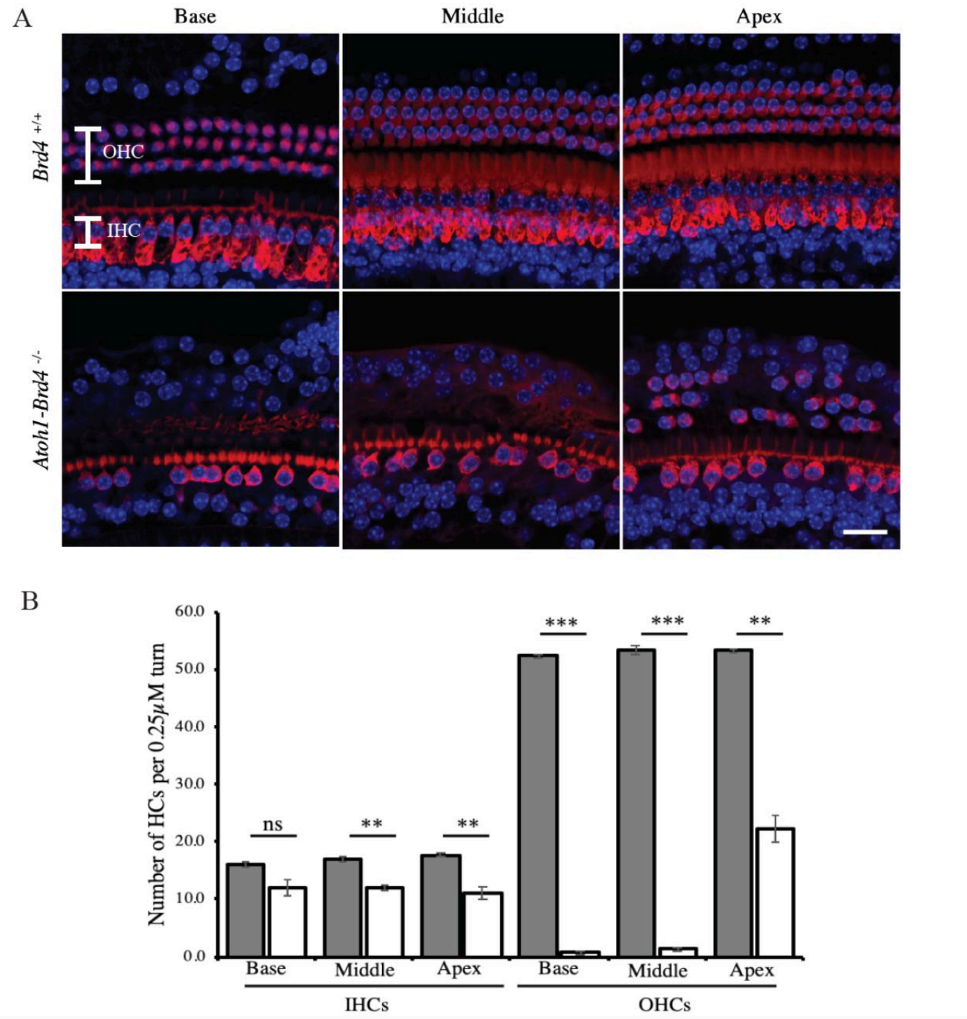

Supplementary Figure 3. Hair cell loss in different regions of cochlea (A) Z-stack of Atoh1-Brd4-/- P21 mouse cochleae immunolabeled with HC marker Myo7a (red) and DAPI (blue) (scale:20μm) showed severe HC loss at P21 in basal, middle and apical regions, with more OHC death compared to IHCs. (B). Graphical representation of the quantification of the number of OHCs and IHCs (per 0.25μm turn of cochlea) of the different regions of cochlea at P21(white, Atoh1-Brd4-/-; grey, Brd4+/+). The error bars indicate the SEM (Standard T-test was performed to determine the significance of the experiment, * $p<0.05$, ** $p< 0.01$, *** $p<0.001$, ns-not significant).
