# Supplementary material for: Bromodomain Protein BRD4 Is Essential for Hair Cell Function and Survival
Source: Front Cell Dev Biol. 2020 Sep 8;8:576654. doi: 10.3389/fcell.2020.576654 (PMC7509448; doi:10.3389/fcell.2020.576654)
Supplement: Supplementary file 3 [file Image_3.pdf]

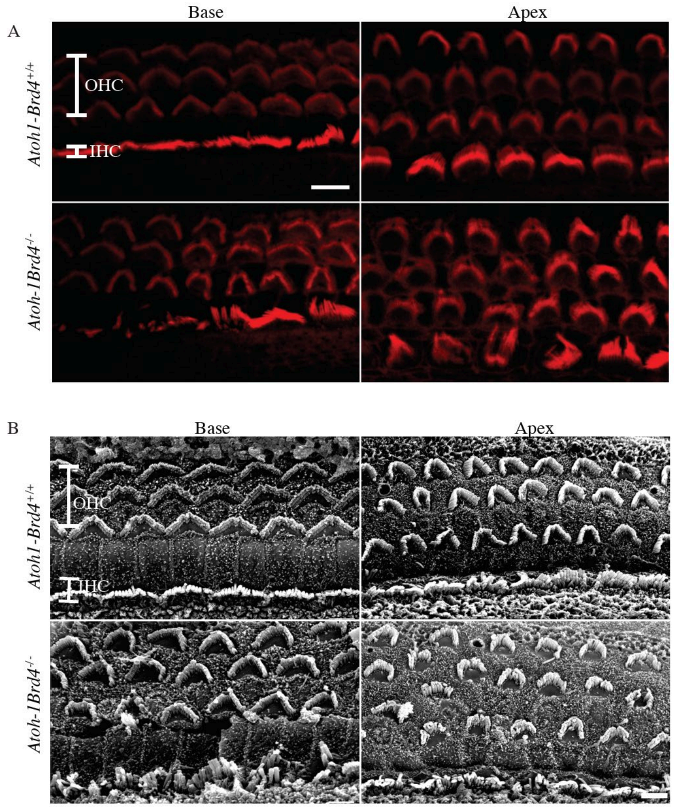

Supplementary Figure 2. Figure 2. Stereocilia degeneration **(A)** Z-stack of mouse cochleae (P8, n=3) counterstained for actin filaments (red) (scale: 10µm) shows degeneration of stereocilia in the basal and apical regions of the cochleae in *Atoh1-Brd4*$^{-/-}$ compared to the control. **(B)** SEM images of P8 (n=2) mouse cochleae processed using the O-T-O-T-O showed show the difference in the structural integrity of the stereocilia between *Brd4*$^{+/+}$ and *Atoh1-Brd4*$^{-/-}$ (Scale: 10µm upper panel) in the basal and apical regions of the cochleae.
